# Stealth and Cationic Nanoliposomes as Drug Delivery Systems to Increase Andrographolide BBB Permeability

**DOI:** 10.3390/pharmaceutics10030128

**Published:** 2018-08-13

**Authors:** Vieri Piazzini, Elisa Landucci, Giulia Graverini, Domenico E. Pellegrini-Giampietro, Anna Rita Bilia, Maria Camilla Bergonzi

**Affiliations:** 1 Department of Chemistry, University of Florence, Via Ugo Schiff 6, Sesto Fiorentino, 50019 Florence, Italy; vieri.piazzini@unifi.it (V.P.); giulia.graverini@stud.unifi.it (G.G.); ar.bilia@unifi.it (A.R.B.); 2 Department of Health Sciences, Section of Clinical Pharmacology and Oncology, University of Florence, Viale Pieraccini 6, 50139 Florence, Italy; elisa.landucci@unifi.it (E.L.); domenico.pellegrini@unifi.it (D.E.P.-G.)

**Keywords:** liposomes, brain delivery, surfactant, cationic liposomes, andrographolide, PAMPA, hCMEC/D3 cells

## Abstract

(1) Background: Andrographolide (AG) is a natural compound effective for the treatment of inflammation-mediated neurodegenerative disorders. The aim of this investigation was the preparation of liposomes to enhance the penetration into the brain of AG, by modifying the surface of the liposomes by adding Tween 80 (LPs-AG) alone or in combination with Didecyldimethylammonium bromide (DDAB) (CLPs-AG). (2) Methods: LPs-AG and CLPs-AG were physically and chemically characterized. The ability of liposomes to increase the permeability of AG was evaluated by artificial membranes (PAMPA) and hCMEC/D3 cells. (3) Results: Based on obtained results in terms of size, homogeneity, ζ-potential and EE%. both liposomes are suitable for parenteral administration. The systems showed excellent stability during a month of storage as suspensions or freeze-dried products. Glucose resulted the best cryoprotectant agent. PAMPA and hCMEC/D3 transport studies revealed that LPs-AG and CLPs-AG increased the permeability of AG, about an order of magnitude, compared to free AG without alterations in cell viability. The caveolae-mediated endocytosis resulted the main mechanism of up-take for both formulations. The presence of positive charge increased the cellular internalization of nanoparticles. (4) Conclusions: This study shows that developed liposomes might be ideal candidates for brain delivery of AG.

## 1. Introduction

The major hindrance in the treatment of brain disorders is the blood–brain barrier (BBB), which prevents the transfer of most drugs, peptides and large molecules across the endothelial cell lining to protect the brain from undesirable side effects. To overcome such problems various approaches are used.

The liposomes offer a promising tool to resolve the low permeability and high selectivity of the BBB.

Liposomes are non-toxic, biocompatible and biodegradable drug carrier systems. Their structure which is composed of phospholipids with an aqueous reservoir allows the encapsulation of a wide variety of hydrophilic and hydrophobic agents [1,2,3,4]. Their phospholipid bilayer structure, similar to physiological membranes, makes them more compatible with the lipoid layer of BBB and increase the permeability of the drug.

Liposomes allow relatively higher intracellular uptake than other particulate systems, due to their sub-cellular size. They are highly studied for the treatment of central nervous system’s pathologies such as infections, cerebral ischemia, brain tumors and neurodegenerative diseases, for instance Parkinson’s and Alzheimer’s [5,6]. Several studies have reported an increased transport across the BBB of encapsulated drugs both through intracerebral and intravenous administration [7].

The surface can be modified with functional ligands to enhance the brain targeting. The functionalized nanoparticles with structures able to interact with targets on the surface of the BBB represents a tool of enormous potentiality to ameliorate the bioavailability and to reduce side effects. Several studies on animal models of Alzheimer’s disease demonstrated the efficacy of functionalized liposomes to cross BBB and ameliorate impaired cognitions [8,9,10].

In a previous research studies, the authors developed solid lipid nanoparticles [11] and polymeric nanoparticles [12] to deliver the andrographolide (AG), a natural compound, through the central nervous system and ameliorate its biopharmaceutical characteristics.

AG is one of the characteristic diterpenoids from *Andrographis paniculata* with a wide spectrum of biological activities, being anti-inflammatory, anticancer, hepatoprotective and antihyperlipidemic. AG is involved in oxidative stress-related pathways implicated in stroke pathogenesis and it protects against ischemic stroke [13]. Furthermore, it has shown protection against damage induced by amyloid-β oligomers in vitro, it reduces amyloid-β levels and tau phosphorylation in mice, it modulates the formation of amyloid plaques and it retrieves spatial memory functions in Alzheimer’s disease transgenic mouse model [14]. The high lipid solubility of AG would permit its penetration of the BBB but its poor water solubility and stability reduces its bioavailability: indeed, these factors are the greatest drawbacks for clinical application [15,16].

In recent years, surfactants such as Tween 80 have been studied for the application in liposomal formulations. The sterically stabilized liposomes exhibited a superior entrapment stability compared with surfactant-free liposomes [17]. The surfactant during preparation of liposomes helps in efficient emulsification resulting in decreasing the size of vesicles and promotes the flexibility of the vesicle to penetrate the biological cell membranes. Tween 80 was also able to enhance liposomes half-life [18] and, in addition, has interesting properties including the formation of a superficial coating on liposomes that can produce “stealth” nanocarriers. Tween 80 can adsorb ApoE, which subsequently binds to its specific LDL receptor by increasing carrier endocytosis at the level of cerebral endothelial cells [4,19,20,21,22]. Finally, this surfactant is also an inhibitor of the P-gp effluent pump [23].

Another approach is the use of the cationic liposomes, able to cross the BBB via absorption-mediated transcytosis [24]. Several studies have shown that these cationic nanocarriers are more efficient vehicles for drug delivery to the brain than conventional, neutral, or anionic liposomes, possibly due to the electrostatic interactions between the cationic liposomes and the negatively charged cell membranes, enhancing nanoparticle uptake. In particular, this kind of liposome interacts with the endothelial cells of microvessels rich in lecithin, which binds positively charged material and induces its cell internalization process through endocytosis [6,24]. Furthermore, the cationic liposomes very easily fuse with cells.

The aim of the present study was the formulation of nano-sized liposomes of AG for brain targeting. Tween 80 alone or in combination with Didecyldimethylammonium bromide (DDAB) were considered to investigate the effects, on chemical and physical aspects, stability, release characteristics, in vitro uptake and permeability of the AG liposomes and to ameliorate the loading and the solubility of AG.

Liposomes were evaluated for various formulation parameters (size, polydispersity, ζ-potential, morphology, chemical and physical stability, in vitro release) and the optimized formulations were studied and characterized with in vitro tests. The ability of liposomes to increase the permeability of AG was evaluated by a Parallel Artificial Membrane Permeability Assay (PAMPA) [25]. Furthermore, the uptake of liposomes as well as their permeability across hCMEC/D3 monolayer cells, as an in vitro BBB model [11,26,27], were considered. Cell viability and cytotoxicity studies were also conducted.

## 2. Materials and Methods

### 2.1. Materials

Egg phosphatidylcholine (Phospholipon 90G) was purchased from Lipoid AG, Cologne, Germany with the support of its Italian agent AVG srl, Milan, Italy. Andrographolide, Cholesterol ≥95%, Didecyldimethylammonium bromide (DDAB, 98%), Coumarin-6 (6C), Fluorescein sodium salt (NaF), Human Serum Albumin (HSA), Phosphate Buffered Saline (PBS 0.01 M) powder (29 mM NaCl, 2.5 mM KCl, 7.4 mM Na_2_HPO_4_·7H_2_O, 1.3 mM KH_2_PO_4_) pH 7.4 and Tween 80 were from Sigma Aldrich, Milan, Italy. Glucose anhydrous and sucrose came from Merck, Darmstadt, Germany. 96-well Multi-Screen PAMPA filter plate (pore size 0.45 μm) were purchased from Millipore Corporation, Tullagreen, Carrigtwohill, County Cork, Ireland. Porcine polar brain lipid was obtained from Avanti Polar Lipids, Inc., Alabaster, AL, USA. All the solvents used (acetonitrile, dichloromethane, dodecane, ethanol, formic acid, methanol) were HPLC grade from Sigma Aldrich, Milan, Italy. Water was purified by Millipore, Milford, MA, USA, Milli-Qplus system. Phosphotungstic acid (PTA) was from Electron Microscopy Sciences, Hatfield, PA, USA.

### 2.2. Preparation of Liposomal Carriers

Stealth liposomes containing Tween 80 (LPs) and cationic liposomes (CLPs) with Tween 80 and DDAB were prepared according to the thin layer evaporation method [28]. For LPs, 160 mg of egg phosphatidylcholine (P90G) and 10 mg of cholesterol (CHOL) were dissolved in dichloromethane. The organic solvent was vacuum evaporated, and the dry lipid film was hydrated by adding 10 mL PBS containing Tween 80 at a concentration of 3% *w*/*v*. The aqueous dispersion was shaken with a mechanical stirrer for 30 min in a water bath at the constant temperature of 37 °C. In order to obtain small unilamellar vesicles from multilamellar vesicles, an ultrasonication probe was used for 10 min (with pulsed duty cycles of ½ s on and ½ s off, amplitude 50%) with the sample in an ice bath to prevent lipid degradation [29]. Finally, a gentle centrifugation of 1 min at 1205× *g* was performed to remove possible metallic particles released by the ultrasonic probe inside the liposomal dispersion [30].

In addition, for CLPs, 10 mg of DDAB were weighted together with P90G and CHOL and then vesicles were prepared by hydrating the dry lipid film with 10 mL PBS containing 3% of Tween 80 [31].

AG-loaded LPs (LPs-AG) and AG-loaded CLPs (CLPs-AG) were prepared with the same method described above, adding 8.5 mg of AG (0.85 mg/mL, corresponding to 5% of the weight of the lipid component) together with P90G, CHOL, DDAB in the case of CLPs-AG and 1–2 mL of methanol with dichloromethane to completely dissolve AG.

Coumarin-6-loaded liposomes (LPs-6C and CLPs-6C) were prepared using the same method, adding 5 mg of the probe (λ_max_ = 444, λ_ex_ = 420 nm, λ_em_ = 505 nm, green), corresponding to 3% of the weight of the lipid component, to the organic phase.

### 2.3. Physical and Morphological Characterization

Liposomes’ hydrodynamic diameter, size distribution and ζ-potential were measured by Light Scattering (LS), using a Zsizer Nano series ZS90 (Malvern Instruments, Malvern, UK) outfitted with a JDS Uniphase 22 mW He-Ne laser operating at 632.8 nm, an optical fiber-based detector, a digital LV/LSE-5003 correlator and a temperature controller (Julabo water-bath) set at 25 °C. Time correlation functions were analyzed by the Cumulant method, to obtain the hydrodynamic diameter of the vesicles (Z_average_) and the particle size distribution (polydispersity index, PdI) using the ALV-60 × 0 software V.3.X provided by Malvern. ζ-potential, instead, was calculated from the electrophoretic mobility, using the Henry correction to Smoluchowski’s equation. The samples were diluted 100-fold in distilled water and an average of three measurements at stationary level was taken. A Haake temperature controller kept the temperature constant at 25 °C.

Liposomes were also analyzed in terms of morphology, shape, and dimensions by the transmission electron microscopy (TEM). The aqueous dispersion was diluted 10-fold in PBS and 5 μL were applied to a carbon film-covered copper grid. Most of the sample was blotted from the grid with filter paper to form a thin film. After the adhesion of liposomes, 5 μL of phosphotungstic acid solution (1% *w/v* in sterile water) were dropped onto the grid as a staining medium and the excess solution was removed with filter paper. Samples were dried for 3 min, after which they were examined with a JEOL 1010 electron microscope and then photographed at an accelerating voltage of 64 kV.

### 2.4. Chemical Characterization of Formulations

The percentage of the AG or 6C entrapped into liposomes in respect to the amount of substances initially used in the liposomal preparation was expressed as encapsulation efficiency (EE%) and calculated using the direct method. Free AG or 6C was removed by means of dialysis. 2 mL of liposomal suspensions were transferred in a dialysis bag (cut-off 3500–5000 Dalton), which was stirred in 1 L of water at room temperature for 1 h [29]. The content of AG or 6C entrapped within liposomes was quantified by HPLC-DAD analysis, respectively after disruption with methanol of purified liposomes (placed in the ultrasonic bath for 30 min) and ultracentrifugation for 10 min at 11,330× *g*.

LC% for liposomal formulations was calculated using the following Equation (1):

(1) $$\;\mathrm{LC}\%=\frac{\mathrm{Total}\;\mathrm{amount}\;\mathrm{of}\;\mathrm{determined}\;\mathrm{drug}}{\mathrm{Weight}\;\mathrm{of}\;\mathrm{liposomes}}\times 100\;$$

The Recovery% was carried out with the same procedure but without initial dialysis and was calculated using the following Formula (2):

(2) $$\;\mathrm{Recovery}\%=\frac{\mathrm{Total}\;\mathrm{amount}\;\mathrm{of}\;\mathrm{determined}\;\mathrm{drug}}{\mathrm{Initial}\;\mathrm{amount}\;\mathrm{of}\;\mathrm{drug}\;\mathrm{loading}}\times 100\;$$

### 2.5. HPLC-DAD and HPLC-FLD Methods

An HP 1100 liquid chromatograph equipped with a DAD detector was used to carry out the quali-quantitative determinations of AG. A 150 mm × 4.6 mm i.d., 5 μm Zorbax Eclipse XDB, RP18 column (Agilent Technologies, Santa Clara, CA, USA) was employed. The mobile phases were (A) CH_3_CN and (B) formic acid/water pH 3.2. Flow rate was 0.8 mL/min and temperature were set to 27 °C. The following gradient profile was utilized: 0–2 min, 5–15% A, 95–85% B; 2–5 min, 15% A, 85% B; 5–7 min 15–50% A, 85–50% B; 7–12 min, 50% A, 50% B; 12–15 min, 50–30% A, 50–70% B; 15–20 min, 30% A, 70% B; 20–25 min, 30–5% A, 70–95% B with equilibration time of 5 min. Injection volume was 10 μL. The UV/vis spectra were recorded in the range 200–800 nm and the chromatograms were acquired at 223 nm.

6C characterization was performed using an HP 1200 liquid chromatograph with Luna RP18 column (4.6 mm × 250 mm i.d., 5 μm) maintained at 25 °C. The mobile phase was composed of (A) CH_3_CN and (B) formic acid/water pH 3.2 with a flow rate of 1 mL/min. The gradient profile was: 0–2 min, 30% A, 70% B; 2–26 min 30–100% A, 70–0% B; 26–29 min 100% A, 0% B; 29–35 min 100–30% A, 0–70% B with post-time of 5 min. Chromatograms were acquired at 444 nm.

An HP 1200 liquid chromatograph equipped with a FLD detector was used for the quantification of NaF probe (λ_ex_ = 460 nm, λ_em_ = 515 nm, green). The column was a Kinetex C18 (4.6 mm × 150 mm i.d., 5 μm) maintained at 27 °C. The mobile phases were (A) CH_3_CN and (B) formic acid/water pH 3.2. Flow rate was 0.8 mL/min and the injection volume was 10 μL. The following gradient profile was utilized: 0–3 min, 20% A, 80% B; 3–23 min, 20–80% A, 80–20% B; 23–25 min 80–100% A, 20–0% B; 25–27 min, 100–20% A, 0–80% B with equilibration time of 5 min.

Diluting stock solutions in CH_3_OH (0.5 mg/mL for AG and 0.1 mg/mL for 6C) and in H_2_O (0.1 mg/mL for NaF), standard solutions were freshly prepared. To quantify each compound, an external standard method was applied using a regression curve and analyses were performed in triplicate. Results were expressed as the mean ± SD of the 3 experiments.

All the compounds showed a linear response: AG from 0.05 to 25 μg/mL, NaF from 0.05 to 46 μg/mL and 6C from 0.515 to 51.5 μg/mL. All the curves had coefficients of linear correlation R^2^ ≥ 0.999.

Progressive dilutions of standard solutions were used to calculate the limit of detection LOD (S/N ≥ 3) and the limit of quantification LOQ (S/N ≥ 10). LOD and LOQ for AG were 2.6 ng and 5.3 ng, respectively.

### 2.6. Stability Studies

The stability of empty and AG-loaded liposomes was studied for one month. Aqueous dispersions were kept at 4 °C and, at fixed time intervals, their physical and chemical stabilities were assayed: physical stability was checked by monitoring sizes, polydispersity index and ζ-potential, while chemical stability was determined by quantification of encapsulated drug by HPLC-DAD analysis. 

The freeze-drying process in the absence of cryoprotectant and in the presence of 1% *w/v* of glucose or sucrose was also considered. Afterwards, lyophilization physical stability was checked for one month at 25 °C.

200 μL of LPs and CLPs dispersions were incubated at body temperature with a solution of human serum albumin (HSA, 40 mg/mL in PBS) for two hours under magnetic stirring to mimic in vivo conditions [32,33]. Physical stability of the formulations was evaluated using Dynamic Light Scattering, by controlling liposomes sizes at regular intervals. 

The yield of the preparation of freeze-dried LPs-AG and CLPs-AG was calculated as the weight of the product obtained after the freeze-drying, compared to the weight of the components used in the reaction (3):

(3) $$\;\mathrm{Yield}\%=\frac{\mathrm{real}\;\mathrm{weight}\;\left(\mathrm{mg}\right)}{\mathrm{teoric}\;\mathrm{weight}\;\left(\mathrm{mg}\right)}\times 100\;$$

### 2.7. In Vitro Release

AG in vitro release from liposomes was performed using a dialysis membrane (cut-off 3000–5000 Dalton) in PBS at 37 °C. Two mL of AG solution (0.85 mg/mL in methanol), LPs and CLPs suspensions were filled in pre-soaked dialysis tubes and placed in 200 mL of release medium using a magnetic stirrer. An aliquot of 1 mL of release medium was removed at pre-determined time intervals and replaced with 1 mL of fresh PBS maintained at 37 °C [34]. AG concentration at different times was calculated using HPLC analyses: the mean of triplicate drug release and standard deviation (mean ± SD, *n* = 3) was used to draw the drug release profiles.

The following Formula (4) was applied to calculate the percentage of AG released in the medium at pH 7.4 at each time interval (0, 30, 60, 120, 240, 360 and 1440 min):

(4) $$\;\%\;\mathrm{drug}\;\mathrm{released}=\frac{\mathrm{drug}\left(t\right)\;\left(\mathrm{mg}\right)}{\mathrm{total}\;\mathrm{drug}\;\left(\mathrm{mg}\right)}\times 100\;$$

To evaluate the kinetics and mechanism of drug release from the liposomes, the Korsmeyer–Peppas model, Hixson Crowell model, Higuchi model, first order and zero order mathematical models were used and the best fitted model was selected based on high regression coefficient (R^2^) value for the release data.

### 2.8. PAMPA Studies

PAMPA studies for LPs-AG and CLPs-AG were carried out using the method previously published [11]. A solution (2% *w*/*v*) of Porcine Polar Brain Lipid (PBL) in n-dodecane was prepped and the mixture was sonicated. PBL solution (5 μL) was added to each donor plate well [16]. Right after the application of the artificial membrane, 250 μL of formulation were added to each donor compartment, whilst the acceptor compartment was filled with PBS/Ethanol solution. Then the drug-filled donor compartment was installed into the acceptor plate. After incubation for 18 h, the donor and acceptor plate samples were withdrawn and analyzed by HPLC-DAD analyses for quantification of AG concentration: 150 μL were taken from both compartments, later diluted with methanol, placed in the ultrasonic bath for 30 min and finally ultra-centrifuged for 10 min at 11,330× *g* (4 °C). The permeability of AG was calculated using the following Formula (5) [35]:

P_e_ = −ln [1 − C_A_(t)/C_equilibrium_]/A × (1/V_D_ + 1/V_A_) × t (5)

where P_e_ is permeability in the unit of cm/s, effective filter area (A) = f × 0.3 cm^2^, where f = apparent porosity of the filter, C_A_(t) = compound concentration in receptor well at time t, V_D_ = donor well volume (mL), V_A_ = receptor well volume (mL), t = incubation time (s), C_D_(t) = compound concentration in donor well at time t, and (6)

C_equilibrium_ = [C_D_(t) × V_D_ + C_A_(t) × V_A_]/(V_D_ + V_A_) (6)

The experiments were performed in triplicate.

### 2.9. hCMEC/D3 Cell Culture

This cell line (Millipore Cat. # SCC066) derives from human temporal lobe micro-vessels isolated from tissue excised during surgery for epilepsy control. Cells were seeded in a concentration of 2.5 × 10^4^ cells/cm^2^ and grown at 37 °C in an atmosphere of 5% CO_2_ in 25 cm^2^ rat tail collagen type I coated culture flasks. EndoGRO^TM^-MV Complete Media Kit (Cat. # SCME004) supplemented with 1 ng/mL FGF-2 (Cat. #GF003) was changed every three days and cells were grown until they were 90% confluent. Cells were passaged at least twice before use. Confluent hCMEC/D3 cells were split by Accumax^TM^ Cell Counting Solution in DPBS. 

### 2.10. 3-(4,5-Dimethylthiazol-2-yl)-2,5-diphenyltetrazolium Bromide (MTT) Assay

To assess cell viability after AG and LPs-AG and CLPs-AG exposure, an MTT assay was performed [36,37]. Cells were seeded in a 24-well plate (6 × 10^4^ cells/cm^2^) pre-coated with Collagen Type I, Rat Tail (Cat. #08–115) and grown at 37 °C in an atmosphere of 5% CO_2_ in EndoGRO^TM^ Basal Medium (EBM-2). When the cells were approximately 70–80% confluent they were incubated with different concentrations of AG (10 and 100 µM), LPs-AG (0.085 and 0.0085 mg/mL) and CLPs-AG (0.085 and 0.0085 mg/mL), obtained by dilution (1:10 and 1:100) of the formulation in EBM-2 for 2, 4 and 24 h. The liposome formulations were previously filtered through 0.4 μm sterile filter units. The medium of each well was separated from the cells and stored for lactate dehydrogenase (LDH) assay, and cells were treated with 1 mg/mL of MTT for 1 h at 37 °C and 5% CO_2_. Finally, DMSO was added to dissolve MTT formation and absorbance was measured at 550 and 690 nm. Cell viability was expressed as a percentage compared to the cells incubated only with EBM-2 (positive control). Triton X-100 was used in the MTT assay as the negative control since its detergent action disrupts the cells.

### 2.11. LDH Assay

Cytotoxicity after AG and liposomes exposure was verified with LDH assay. The medium resulting from incubation of AG and liposomes with cells was centrifuged (250× *g*, 10 min at RT) and the supernatant separated from the deposited cells in each well. This centrifugation process allowed us to remove any waste and cellular debris as well as AG and liposomes. The release of LDH into culture supernatants was detected by adding catalyst and dye solutions of a Cytotoxicity Detection Kit (LDH) (Roche Diagnostics, Indianapolis, IN, USA). The absorbance values were recorded at 490 nm and 690 nm. Cytotoxicity was expressed as a percentage compared to the maximum LDH release in the presence of triton X-100 (positive control). EBM-2 was used as negative control since no cytotoxicity was detected in such conditions.

### 2.12. hCMEC/D3 Cell Culture for Transwell Permeability Studies

High density pore (2 × 10^6^ pores/cm^2^) transparent PET membrane filter inserts (0.4 μm, 23.1 mm diameter, Falcon, Corning BV, Amsterdam, Netherlands) were used in 6-well cell culture plates (Falcon, Corning, Amsterdam, Netherlands) for all transcytosis assays. The transparent PET membrane filter inserts were coated with rat tail collagen type I at a concentration of 0.1 mg/mL and incubated at 37 °C for 1 h prior to cell barrier coating. Inserts were subsequently washed with PBS and incubated for 1 h, after which PBS was removed and replaced with the assay medium. The inserts were calibrated for at least 1 h with assay medium at 37 °C. Optimum media volumes were calculated to be 1 mL and 1.2 mL respectively for apical and basolateral chambers. The transwell inserts were calibrated with assay medium for 1 h, then the medium was removed and hCMEC/D3 cells were seeded onto the apical side of the inserts at a density of 6 × 10^4^ cells/cm^2^ in 1 mL assay media. 1.2 mL of fresh medium was added to the basolateral chamber. The assay medium was changed every 3 days following transwell apical insert seeding with hCMEC/D3. For seven days, cells were grown to confluence. hCMEC/D3 monolayers were used as a permeability assay for AG and AG-loaded liposomes. Fluorescein sodium salt (NaF) was considered at a concentration of 10 μg/mL as an integrity control marker with a known permeability coefficient (P_app_) for this cell line [19]. The integrity of monolayer cells was confirmed also by observation of cultures under phase-contrast microscopy or under bright-field optics using of transparent membranes. The image was observed using an inverted microscope (Olympus IX-50; Solent Scientific, Segensworth, Fareham, UK) with a low-power objective (20X). The images were digitized using a video image obtained with a CCD camera (Diagnostic Instruments Inc., Sterling Heights, MI, USA) controlled by software (InCyt Im1TM; Intracellular Imaging Inc., Cincinnati, OH, USA).

For permeability studies, AG (10, and 100 µM), LPs-AG and CLPs-AG (0.085 mg/mL, corresponding to AG 240 μM) obtained by dilution 1:10 of the formulation in EBM-2 were tested and incubated for 1, 2, 3 and 4 h in the apical donor compartment. At the end of the incubation, the amount of NaF and AG were quantified both in apical and basolateral compartments by HPLC-FLD or HPLC-DAD method. In the case of the formulation, EBM-2 was diluted with methanol and placed in the ultrasonic bath for 30 min and then ultra-centrifuged for 1 h at 11,330× *g* (4 °C). The apparent permeability coefficients (P_app_) of free AG and AG encapsulated in LPS and CLPs were calculated according to the Equation (7):

P_app_ (cm/s) = V_D_/(A·M_D_) × (ΔM_R_/Δt) (7)

where: V_D_ = apical (donor) volume (cm^3^), M_D_ = apical (donor) amount (mol), ΔM_R_/Δt = change in amount (mol) of compound in receiver compartment over time.

The recovery for AG and NaF was calculated according to the Equation (8) [19]:

Recovery (%) = C_Df_·V_D_ + C_Rf_ ·V_R_/(C_D0_·V_D_) × 100 (8)

where C_Df_ and C_Rf_ are the final compound concentrations in the donor and receiver compartments, C_D0_ is the initial concentration in the donor compartment and V_D_ and V_R_ are the volumes in the donor and receiver compartments, respectively. All experiments were performed at least in triplicate.

### 2.13. Cellular Uptake of LPs-6C and CLPs-6C

For the evaluation of the intracellular content of 6-Coumarin, hCMEC/D3 cells (1 × 10^4^) were exposed for 2 h to the LPs-6C and CLPs-6C loaded with 0.5 mg/mL of 6C and diluted 1:100 into EBM-2, and to a saturated solution of fluorescent probe. To elucidate the endocytic uptake mechanisms, these experiments were carried out in presence/absence of endocytic inhibitors. Control cells were exposed to liposomal formulations without any agent pre-treatment and their uptake was assumed to be 100%. A second group of cells was pre-treated with 15 μM chlorpromazine for 30 min followed by incubation with liposomes. A third group of cells was pre-treated with 25 μM of indomethacin for 30 min; and, finally, a fourth group of cells was maintained at 4 °C during the LPs-6C and CLPs-6C exposure to observe the effect of low temperature, a general metabolic inhibitor.

At the end of the treatments, the amount of 6C was quantified on cellular lysate by HPLC. For control cells and cells maintained at 4 °C during exposure, a morphological evaluation of cellular uptake was also performed: hCMEC/D3 cells were cultured on histological slides, treated as described above, fixed in 4% formaldehyde in 0.1 mol/L phosphate buffer, pH 7.4, for 10 min then stained with Fluoro scheld with DAPI (Sigma, Milan, Italy) to display the nucleus and observed by fluorescence microscopy (Labophot-2 Nikon, Tokyo, Japan). Ten photomicrographs were randomly taken for each sample. 

Cellular uptake was investigated by confocal microscopy Nikon Eclipse Ti using liposomes labeled with 6C, with S Fluor 20x, NA = 0.75 high pressure Hg vapor lamp (Intensilight, Nikon, Tokyo, Japan).

Filter set: excitation 365 nm emission 400 nm hi-pass DAPI, excitation 485 nm emission 524 nm 6Co and CCD camera: Coolsnap HQ^2^, Princeton instruments, Trenton, NJ, USA, 1392 × 1040, 6.45 um square pixels.

### 2.14. Statistical Analysis

The experiments were repeated three times and results expressed as a mean ± standard deviation. Statistical significance of hCMEC/D cell viability and cellular uptake was analyzed using one-way ANOVA followed by the post hoc Tukey’s w-test for multiple comparisons. All statistical calculations were performed using GRAPH-PAD PRISM v. 5 for Windows (GraphPad Software, San Diego, CA, USA). A probability value (*p*) of <0.05 was considered significant.

## 3. Results and Discussion

### 3.1. Preparation and Characterization of Liposomes

LPs were prepared by using P90G, CHOL and Tween 80. This compound was selected as a coating agent to increase the stability of the formulation, to produce “stealth” nanovesicles and to promote endocytosis of the carrier at the level of cerebral endothelial cells [23]. Various ratios of the two lipid constituents were tested to obtain small sizes, good polydispersity and favorable ζ-potential. In particular, the ratios P90G:CHOL 18:1, 16:1, 14:1, 12:1, and 10:1 were considered. The best ratio resulted to be 16:1, corresponding to 160 mg of P90G and 10 mg of CHOL. Then, 3% *w/v* of Tween 80 was added (LPs). Furthermore, different sonication times were tested to optimize LPs physical characteristics. The selected conditions consisted in two cycles of 5 min of sonication, each including 0.5 s of sonication alternating with 0.5 s of pause, as reported in the materials and methods section. LPs were nanosized unilamellar vesicles, with a PdI less than 0.25 and a ζ-potential, around −20 mV (Table 1), confirming a homogeneous and stable dispersion. LC% was 2.28% ± 0.22. The mean vesicle sizes and the width of the particle distribution are important parameters as they govern physical stability and permeation through BBB [38]. Moreover, the vesicles sizes highly affected the interaction of the liposomes with the hCMEC/D3 cellular model [39,40].

**Figure 1:**
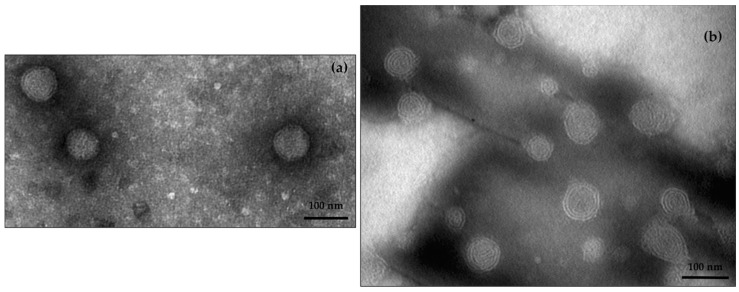
TEM images of LPs-AG (**a**) and CLPs-AG (**b**) (scale 100 nm).

AG does not influence the stability of the formulation (Table 1); when LPs were loaded with AG, LPs-AG showed the same physical parameters as LPs. The electron microscope analysis confirmed the liposomal structure; the results evidenced the presence of spherical vesicles, with a defined phospholipid bilayer, well separated, due to the presence of the surfactant that prevents agglomeration, and with dimensions around 100 nm, confirming the DLS results (Figure 1a). 

Next, LPs were functionalized with positive surface charges by using DDAB (cationic liposomes, CLPs) in the same amount of CHOL (10 mg in the total formulation). In this case, ζ-potential resulted positive, indicating the presence of positive charges on the surface of the carrier.

Then, CLPs loaded with 8.5 mg of AG (corresponding to 5% of the weight of the lipid component) were prepared (CLPs-AG); the presence of DDAB and AG did not modify the physical characteristics of the formulation (Table 1). LC% was 3.08% ± 0.21. TEM analysis showed well separated spherical shape vesicles, with a distinct phospholipid bilayer (Figure 1b).

The compound 6-Coumarin (6C), a lipophilic fluorescent dye, was incorporated into liposomes to investigate the ability of nanoparticles to penetrate into hCMEC/D3 cells, as BBB-model, and to elucidate trans-endothelial transport in vitro. The preparation of fluorescent liposomes was performed as reported for LPs and CLPs, by adding 6C (0.5 mg/mL) to the organic phase. Their physical and chemical parameters are shown in Table 1. The same dimensions of the two types of liposomes, was very important to interact with the HCMEC/D3 equally [39]. LPs-6C and CLPs-6C resulted larger but useful for a parenteral administration. AG and 6C are lipophilic compounds and there are inserted in the bilayer, but the effect on the sizes of nanoparticles is different due to their unlike chemical structure. The high ζ-potential of all formulations is indicative of their stability, as also supported by stability studies.

### 3.2. Stability Studies

**Figure 2:**
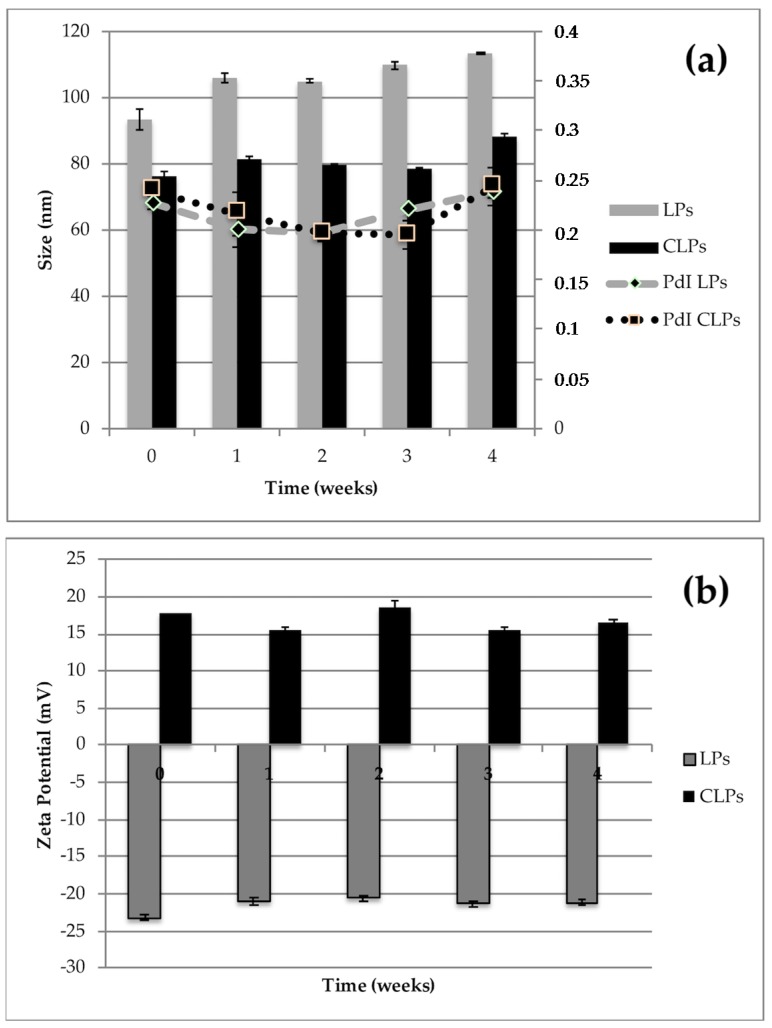
Particle size, polydispersity index (PdI) (**a**) and zeta-potential (**b**) of LPs-AG and CLPs-AG as dispersion after one-month storage at 4 °C. (Data displayed as mean ± SD; *n* = 3).

Liposomes stability was evaluated both as a colloidal dispersion and in the freeze-dried form. The ability of the aqueous dispersions to maintain their physicochemical properties in terms of particle size, PdI, surface charge and drug entrapment was assessed after 1-month storage at 4 °C and the Light Scattering analyses were performed to control the stability over time. No significant changes were observed in physical parameters of empty or LPs-AG and CLPs-AG dispersions (Figure 2). The presence of non-ionic surfactant is expected to reduce the agglomeration between liposomes via steric repulsion. Also, the presence of DDAB on the surface of liposomes prevented the aggregation and the precipitation of the vesicles and increased the systems stability. In addition, the entrapment efficiency remained constant, around 45%.

The major limitation to liposomes use is due to their physical and chemical instability, when the aqueous suspension is stored for an extended period. The poor stability in an aqueous medium forms a real obstacle against the clinical application of nanoparticles.

To improve the physical and chemical stability, water needs to be removed. Freeze-drying is a good technique to enhance the chemical and physical stability of formulations over prolonged periods.

The stability of LPs-AG and CLPs-AG with time was evaluated also in the freeze-dried form. However, the freezing process of the sample might cause problems of possible structural and/or functional damages of the system, and/or subsequent difficulties in sample re-solubilization, due to particle aggregation phenomena. The addition of cryoprotectants improves the quality of the dehydrated product, decreases particle aggregation phenomena and allows to obtain an easier re-dispersion of the freeze-dried product.

Therefore, to estimate the effect of the presence and type of cryoprotectant, empty, LPs-AG and CLPs-AG formulations were freeze-dried with and without sucrose or glucose (1% *w*/*v*). After the lyophilization process, all formulations were dispersed in PBS and analyzed by DLS and ELS (Table 2). As shown in Table 2, the drying process produced an increase in terms of size and PdI, respect to the values reported before the freeze-drying process (Table 1). However, all liposomes maintained characteristics suitable for parenteral administration. The best freeze-drying process was obtained in the presence of glucose both for LPs-AG and CLPs-AG. The EE% remained almost constant around 43%. The yield % of the preparation process was also calculated, in this case without addition of the cryoprotectant, and resulted 69.5% ± 0.1 for LPs and 71.2% ± 0.1 for CLPs (mean ± SD; *n* = 3). After a month of storage at 25 °C the freeze-dried product retained the starting characteristics.

A drawback to the use of nanocarriers, in particular cationic nanocarriers, for brain delivery is their binding to serum proteins that attenuates their surface charge. Therefore, the stability of LPs-AG and CLPs-AG in presence of human serum albumin (HSA) at physiological concentration was also tested. After 2 h of incubation, DLS analyses confirmed that sizes were not affected by the presence of HSA and therefore revealed coexistence of free serum proteins and optimized nanocarrier without any protein corona effect (Table 3).

### 3.3. In Vitro Release

**Figure 3:**
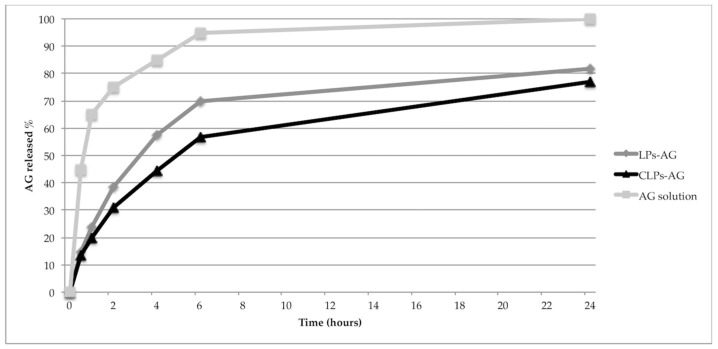
In vitro release profiles of LPs-AG and CLPs-AG in PBS. (each data point represents the average of three samples).

AG in vitro release at 37 °C from LPs-AG and CLPs-AG was evaluated for 24 h by using a dialysis bag and PBS as receptor medium to mimic sink conditions. The release profiles of AG from AG solution, LPs-AG and CLPs-AG were reported in Figure 3. The result indicated that the release of AG from methanol solution through the dialysis membrane was much faster, with a fast release during the first 2 h and approximately 100% of the drug released within 6 h. In contrast, the immediate release of the drug (burst effect) does not occur in the case of LPs-AG and CLPs-AG. The percentages of AG released from LPs and CLPs were gradual: only 56.8% and 69.7% of drug was released within 6 h, respectively. The percentages rose to 83.5% and 77.4%, after 24 h, respectively. The almost linear and gradual trend of the release indicated that the liposomal systems can release AG for prolonged periods and in greater quantities compared to the saturated aqueous solution, were the solubility of AG resulted very low (0.05 mg/mL). Optimized liposomal formulations are able to solubilize 0.85 mg/mL of drug.

Different theoretical models were considered to examine the nature of release. The drug release mechanism was defined by fitting AG release data with various kinetics models. By comparing the regression coefficient values, the Higuchi model (R^2^ = 0.8366 and 0.9264, respectively, Table 4) resulted as the best to describe the kinetics of these two types of liposomes. Thus, the liposomal membrane disruption controlled the release mechanism [41].

Due to the very low solubility of AG in water and the related problems of bioavailability, both the liposomal formulations allow the administration of a high amount of solubilized molecule according to the requirements for parenteral preparations.

### 3.4. PAMPA Study

Parallel Artificial Membrane Permeability Assay (PAMPA) was performed to estimate passive transcellular permeability. It is a non-cell-based permeability model because it lacks transporter- and pore-mediated permeability, but is considered robust, reproducible and it results in a helpful complement to the cellular permeability model for its speed, low cost and versatility, and readily provides information about passive transport permeability.

AG is a molecule with low BBB permeability (effective permeability, P_e_ value of 0.49 ± 0.16 × 10^−6^ cm/s [11] and therefore liposomal formulation could represent a useful tool to improve its permeation. P_e_ of AG-loaded liposomes resulted as increased, in particular 3.94 ± 0.60 × 10^−6^ cm/s for LPs-AG and 3.87 ± 0.36 × 10^−6^ cm/s for CLPs-AG. These values confirmed that LPs-AG and CLPs-AG increased the permeability of the drug, of about an order of magnitude, compared to the aqueous solution.

Though this test does not discriminate the different behavior of the two systems because the artificial membrane fails to mimic all properties of a cell, a mechanism of permeation through the artificial membrane was hypothesized. An interaction between the phospholipid bilayer, which is a flexible system, with the lipid that covers the artificial membrane, similar to one of mechanisms of liposome-cell interaction.

### 3.5. MTT and LDH Assays

**Figure 4:**
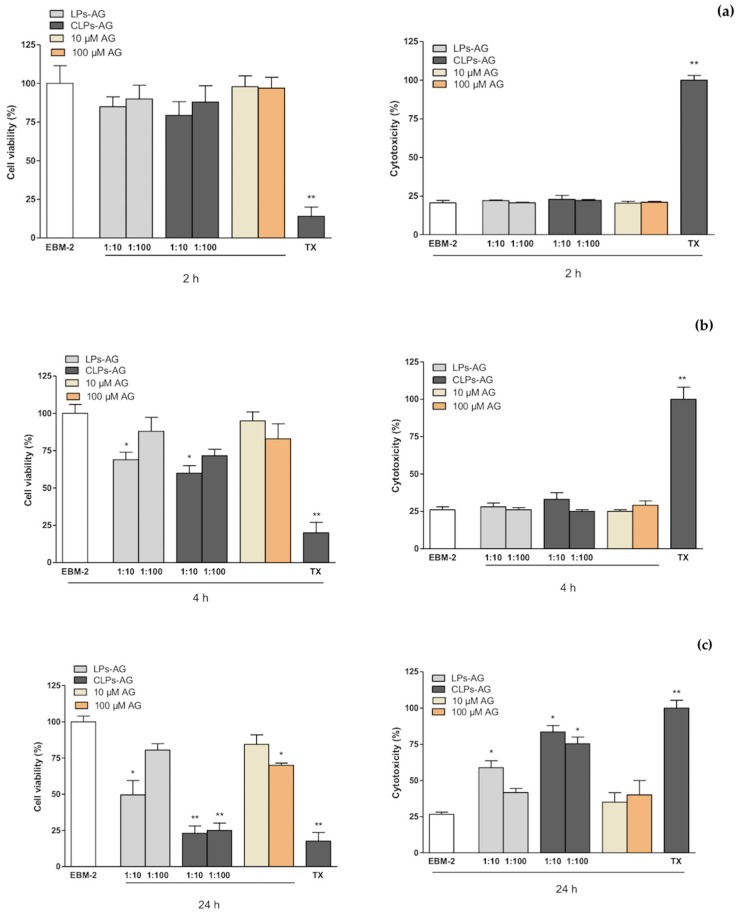
hCMEC/D3 cell viability evaluated by MTT assay (left panel) and cytotoxicity by LDH assay (right panel) when exposed for 2 h (**a**), 4 h (**b**) and 24 h (**c**) to AG (10 and 100 µM) or LPs-AG and CLPs-AG (0.0085 and 0.085 mg/mL). Data is expressed as percentage of control (EBM-2 medium) and Triton-X (TTX) which represent, respectively, the maximum cell viability and cell cytotoxicity. Values represent the mean ± SEM of at least three experiments performed in triplicate. * *p* < 0.05 and ** *p* < 0.01 vs. EBM-2 alone.

MTT and LDH assays were performed in the hCMEC/D3 cell line to evaluate the effect of AG and LPs-AG and CLPs-AG in cell viability and cytotoxicity, and permeability studies were also conducted in transwell devices using the same cell line. The in vitro cytotoxicity of the developed LPs-AG and CLPs-AG and AG was assessed by cell viability determination and membrane integrity evaluation using the hCMEC/D3 cell line in MTT and LDH assays, respectively (Figure 4). When cells were exposed to different concentrations of AG (10 and 100 µM) and LPs-AG and CLPs-AG (0.0085 and 0.085 mg/mL) for 2 (Figure 4a) and 4 h (Figure 4b), no significant changes were observed in MTT metabolization, except for LPs-AG and CLPs-AG (0.085 mg/mL) or LDH release when compared to cells exposed to the EBM-2 medium alone, indicating that AG and LPs-AG and CLPs-AG affected neither the metabolic activity of the cells nor the membrane integrity at these time points. On the other hand, when the cells were incubated for 24 h with AG at a dose of 100 µM, LPs-AG (0.085 mg/mL) and CLPs-AG (0.0085 and 0.085 mg/mL) we observed a significant reduction of cell viability and an increase in cytotoxicity compared to the control (Figure 4c).

### 3.6. BBB Permeability Studies

hCMEC/D3 brain microvascular endothelial cell line is a model of human BBB utilized to study the drug transport mechanisms [18,42]. The cells retain the expression of most transporters and receptors expressed in vivo in the human BBB. hCMEC/D3 apparent permeability coefficient (P_app_) correlates well with in vivo permeability data, and therefore permeability studies were performed to predict the permeability of free AG and LPs-AG and CLPs-AG across the BBB. NaF was used as the negative control and its P_app_ was determined during all transport experiments to monitor the integrity of the cell layer. This aspect was also been checked by phase-contrast microscopy [11].

**Figure 5:**
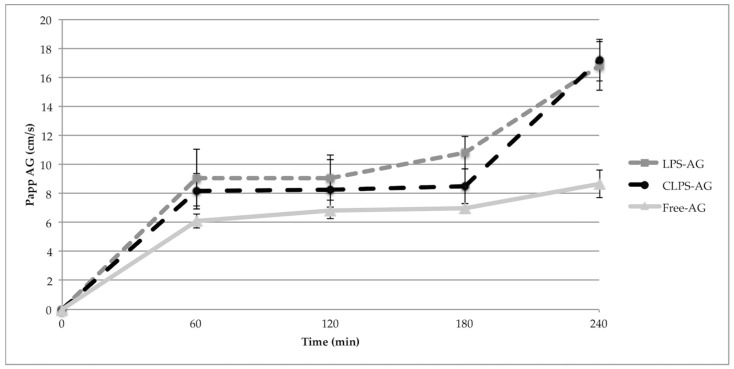
The apparent permeable coefficient of different liposomal formulations for different treatment time in the in vitro BBB model. (Data represent means ± S.D, *n* = 3).

P_app_ of NaF was 8.27 ± 1.81 × 10^−6^ cm/s, in agreement with the literature values [11,12,19]. This value remained constant during the permeability assay, and demonstrates the confluence of the monolayer and assesses the tight junction integrity. P_app_ of LPs-AG and CLPs-AG were only slightly higher than the P_app_ values of the free AG for the first 3 h. However, at 4 h, the P_app_ value for both LPs-AG and CLPs-AG was significantly higher (about double) than that of the free AG (P_app_ of AG: 8.67 ± 0.95 × 10^−6^ cm/s; P_app_ LPs-AG: 16.8 ± 1.70 × 10^−6^ cm/s; P_app_ CLPs-AG: 17.2 ± 1.43 × 10^−6^ cm/s). The amount of AG that permeated when loaded into liposomes increased about 200 times in respect to the free molecule, as confirmed by the increase of the P_app_ values during the time reported in Figure 5. AG transport across the cell was in a time-dependent manner. The obtained P_app_ data are useful for in vitro prediction, as confirmed by the recovery value, which was above 80% in all experiments. The data agrees with PAMPA results. 

A combination assay of PAMPA and unidirectional (apical to basal) hCMEC/D3 permeability model can synergistically provide invaluable permeability/absorption assessment of AG. The P_app_ values are greater than those of P_e_, due to the presence of an active endocytic mechanism in addition to a passive one, as seen by following uptake studies.

### 3.7. Liposome Uptake by hCMEC/D3 Cells

**Figure 6:**
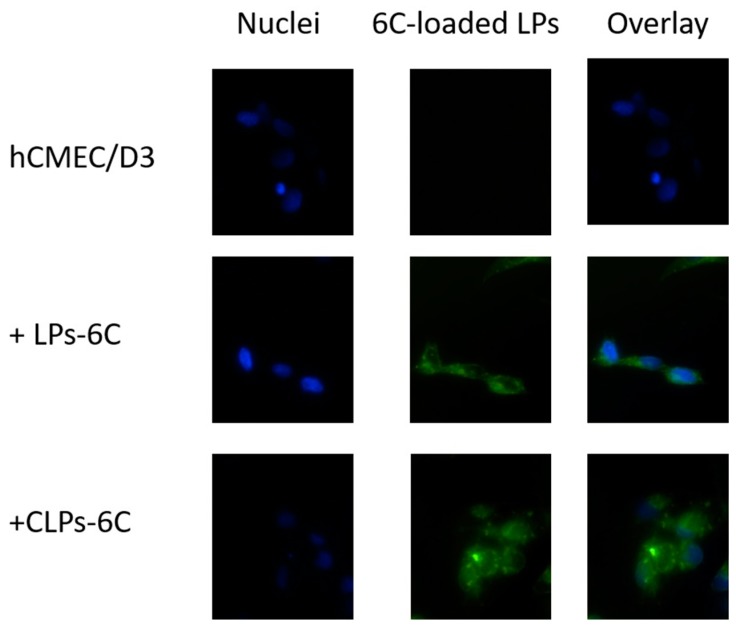
Cellular uptake of LPs-6C and CLPs-6C by hCMEC/D3 cells after 2 h incubation at 37 °C. Images of nuclei stained with DAPI (blue), 6-Coumarin (green) and their overlay. Scale bar: 20 μm.

Figure 6 shows the fluorescence images of hCMEC/D3 cells treated with LPs-6C and CLPs-6C after 2 h of incubation. The images revealed that the probe was internalized and LPs-6C and CLPs-6C were punctually concentrated in intracellular vesicles (endosomes or lysosomes) which can be related to their endocytic mechanism of uptake [42]. Besides their cytoplasmic location, green fluorescence indicates that nanoparticles were also transported to the perinuclear area. This is an important finding for pharmaceutical drug delivery research, since the nucleus is the target site for several drugs [43].

Concerning the uptake of liposomes by hCMEC/D3 cells, it was different for both liposomes LPs-6C and CLPs-6C: 6.4% and 14.0%, respectively. The result proves that the internalization ability of liposomes increases in the presence of positive charge on the bilayer that improves the binding affinity between carrier and cellular membrane [31].

**Figure 7:**
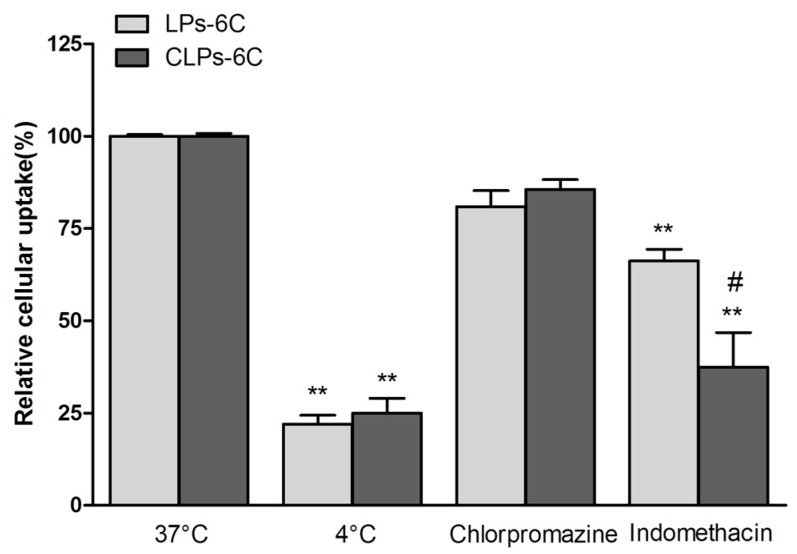
Effect of the temperature (4 °C) and different inhibitors on hCMEC/D3 cell internalization pathways of LPs-6C and CLPs-6C after 2 h incubation at 37 °C. (Data represent the mean ± standard deviation (*n* = 3). Bars represent the mean ± SD of at least 6 experiments ** *p* < 0.01 vs. corresponding liposome at 37 °C; ^#^ *p* < 0.05 CLPs-6C vs. LPs-6C. (ANOVA + Tukey’s test).

Furthermore, a group of cells was maintained at 4 °C in the presence of LPs-6C and CLPs-6C, to observe the effect of low temperature, a general metabolic inhibitor. The fluorescence of cells incubated with 6C loaded liposomes in the absence of any inhibitor was considered as 100%, while the fluorescence after incubation in the presence of inhibitors was expressed as a relative percentage compared to the cells without inhibitor. As shown in Figure 7, a significant reduction (about 80%) in hCMEC/D3 cell uptake efficiency was observed at 4 °C for all two formulations as compared with that at physiological temperature, suggesting that their uptake relied on an energy-dependent pathway and it was mediated by endocytosis [44].

To elucidate the endocytic uptake mechanisms, the experiments were also carried out in the presence of endocytic inhibitors, such as chlorpromazine, a clathrin blocker, and indomethacin, a caveolin-dependent endocytosis inhibitor.

Liposomes could be internalized into cells by different mechanisms, according to the type of cell, composition, surface charge, and size of the liposome [45,46,47]. In our study, the cellular association of the liposomes was significantly influenced by indomethacin, with a reduction in cellular association of about 44% and 63% for LPs-6C and CLPs-6C, respectively (Figure 7). Caveolae-mediated endocytosis is involved in the uptake of liposomes. However, it did not completely inhibit active uptake of the nanoparticles when compared with the results at 4 °C, confirming that cellular uptake of liposomes involved more that on an energy-dependent pathway. In fact, as evidenced in the Figure 7, an uptake reduction of 15% for LPs-6C and 20% for CLPs-6C was observed in the presence of chlorpromazine, even if less pronounced than with indomethacin. This indicates that clathrin-mediated endocytosis is also a mechanism involved in the uptake.

Therefore, the preferential mechanism of the liposomes uptake by hCMEC/D3 cells was found to be caveolae-mediated endocytosis, with a greater effect of inhibitors in the case of CLPs-6C.

## 4. Conclusions

Based on the obtained results in terms of size, homogeneity, ζ-potential and EE%, both optimized liposomal formulations of AG are suitable for parenteral administration. The systems showed excellent chemical and physical stability during a month of storage as suspensions or freeze-dried products. The optimized liposomes enhanced solubility and cellular permeability of AG, as demonstrated by in vitro tests with PAMPA and hCMEC/D3 cells. Both carriers increase the permeation of AG into the cell without alterations in cell viability and monolayer integrity. The presence of positive charge elevated the cellular internalization of liposomes. Uptake experiments suggest an energy-dependent pathway as a possible transport mechanism across the hCMEC/D3 monolayer, with caveolae-mediated endocytosis, in particular, being the main mechanism.

## Figures and Tables

**Table 1:** Physical characterization of empty, andrographolide (AG) and coumarin-6 (6C) loaded liposomes.

Sample	Size (nm)	PdI	ζ-Potential	EE%	Recovery%
LPs	80.2 ± 3.6	0.22 ± 0.03	−20.4 ± 4.1	-	-
CLPs	84.6 ± 8.1	0.23 ± 0.02	20.7 ± 4.7	-	-
LPs-AG	96.4 ± 9.5	0.23 ± 0.03	−22.8 ± 1.2	44.7 ± 3.2	91.1 ± 5.3
CLPs-AG	82.1 ± 9.3	0.25 ± 0.01	20.3 ± 3.7	47.5 ± 3.3	94.9 ± 4.7
LPs-6C	193.1 ± 3.0	0.21 ± 0.02	−27.4 ± 0.4	46.0 ± 1.4	71.2 ± 4.2
CLPs-6C	197.1 ± 1.4	0.27 ± 0.03	31.1 ± 0.6	63.1 ± 0.1	80.6 ± 5.0

LPs: liposomes with Tween 80, CLPs: liposomes with Tween 80 and DDAB. Data displayed as mean ± SD; *n* = 3.

**Table 2:** Physical parameters of andrographolide loaded liposomes, after the freeze-drying process with and without cryoprotectant, 1% *w*/*v* of sucrose or glucose.

LPs-AG	No Cryoprotector	Glucose	Sucrose
Size (nm)	148.8 ± 1.4	135.0 ± 0.9	147.5 ± 1.2
PdI	0.32 ± 0.03	0.25 ± 0.02	0.35 ± 0.01
ζ (mV)	−21.3 ± 0.9	−19.4 ± 1.1	−18.5 ± 1.0
**CLPs-AG**			
Size (nm)	144.6 ± 2.2	131.3 ± 5.1	149.3 ± 1.2
PdI	0.38 ± 0.02	0.28 ± 0.01	0.29 ± 0.01
ζ (mV)	+28.6 ± 0.9	+27.0 ± 0.8	+26.5 ± 0.9

LPs-AG: liposomes with Tween 80 loaded with AG, CLPs-AG: liposomes with Tween 80 and DDAB loaded with AG. Data displayed as mean ± SD; *n* = 3.

**Table 3:** Physical stability of LPs-AG and CLPs-AG in presence of human serum albumin.

	LPs-AG		CLPs-AG	
Time	Size (nm)	Pd	Size (nm)	Pd
**0**	94.8 ± 2.4	0.23 ± 0.02	76.4 ± 1.2	0.24 ± 0.01
**30’**	103.8 ± 2.0	0.39 ± 0.01	82.9 ± 0.5	0.41 ± 0.02
**1 h**	97.2 ± 3.2	0.39 ± 0.02	83.5 ± 4.8	0.40 ± 0.01
**2 h**	99.1 ± 5.1	0.39 ± 0.01	81.9 ± 3.4	0.43 ± 0.02

LPs-AG: liposomes with Tween 80 loaded with AG, CLPs-AG: liposomes with Tween 80 and DDAB loaded with AG. Data displayed as mean ± SD; *n* = 3.

**Table 4:** Regression coefficient (R^2^) obtained in different kinetics models for AG release from LPs-AG and CLPs-AG.

Release Kinetics	LPs-AG	CLPs-AG
Zero order	0.5722	0.7079
First order	0.7685	0.8816
Korsmeyer-Peppas	0.4552	0.4980
Hixson	0.7033	0.8292
Higuchi	0.8366	0.9264

LPs-AG: liposomes with Tween 80 loaded with AG, CLPs-AG: liposomes with Tween 80 and DDAB loaded with AG.
